# TGF‐β activity in acid bone lysate adsorbs to titanium surface

**DOI:** 10.1111/cid.12734

**Published:** 2019-02-28

**Authors:** Franz Josef Strauss, Francesca Di Summa, Alexandra Stähli, Luiza Matos, Fabiola Vaca, Guenther Schuldt, Reinhard Gruber

**Affiliations:** ^1^ Department of Oral Biology Medical University of Vienna Vienna Austria; ^2^ Department of Conservative Dentistry, School of Dentistry University of Chile Santiago Chile; ^3^ Department of Periodontology, School of Dental Medicine University of Bern Bern Switzerland; ^4^ Department of Periodontics University of Southern Santa Catarina Grande Florianopolis Brazil

**Keywords:** bone allograft, bone regeneration, bone resorption, osseointegration

## Abstract

**Objectives:**

Osteoblasts lay down new bone on implant surfaces. The underlying cellular mechanism and the spatio‐temporal mode of action, however, remain unclear. It can be proposed that growth factors released upon acidification by osteoclasts adsorb to the implant surface and control the early stages of osseointegration.

**Methods:**

To simulate bone lysis by osteoclasts, titanium discs were exposed to acid bone lysate (ABL) followed by vigorous washing and seeding of oral fibroblasts. The expression of TGF‐β target genes interleukin 11 (IL11) and NADPH oxidase 4 (NOX4) was evaluated by reverse transcriptase polymerase chain reaction and IL11 ELISA. TGF‐β signaling activation was assessed via Smad2/3 immunofluorescence. The impact of ABL on osteogenic differentiation was determined with murine ST2 mesenchymal stromal cells.

**Results:**

We report here that ABL‐conditioned titanium discs, independent of turned or rough surface, increased the expression of IL11 and NOX4. This increase was blocked by the TGF‐β receptor 1 antagonist SB431542. Further support for the TGF‐β signaling activation came from the translocation of Smad2/3 into the nucleus of oral fibroblasts. Moreover, titanium discs exposed to ABL decreased alkaline phosphatase and osteopontin in ST2 cells.

**Conclusions:**

These in vitro findings suggest that titanium can adsorb TGF‐β from ABLs. The data provide a strong impetus for studies on the protein adsorption on implant surfaces in vitro and in vivo, specifically for growth factors including bone‐derived TGF‐β during successful and failed osseointegration.

## 1 INTRODUCTION

Osseointegration of dental implants is a sequential process that is initiated by drilling the implant bed. The translation of primary mechanical stability into a secondary one is a sequential process initiated by the catabolic action of osteoclasts resorbing the injured necrotic bone.^1^, ^2^ Approximately 1 week after implant placement, the anabolic action of osteoblasts takes over forming new woven bone on the surface of the previously resorbed pristine bone and also on the surface of the titanium implant.^1^, ^3^, ^4^ Woven bone is later replaced by lamellar bone that undergoes modeling for functional adaptation and remodeling for renewal upon fatigue damage.^5^, ^6^ While the later stages of osseointegration, that represent bone remodeling have been thoroughly investigated, the molecular mechanisms supporting the early stages of osseointegration are only beginning to be investigated.^7^

Bone formation requires a solid osteoconductive surface and mesenchymal cells that later undergo osteogenic differentiation,^8^ independent of whether it is fracture healing,^9^ graft consolidation,^10^ or osseointegration of dental implants.^1^ Serum components adsorbing to the implant surface are thought to provide anchorage for mesenchymal progenitors.^11^ The role of growth factors and other molecules released from the bone surface either spontaneously^12^ or upon the resorptive activity of osteoclasts,^13^ however, is still unknown. Considering that the rapid loss of primary stability^14^ is caused by bone‐resorbing osteoclasts,^1^, ^2^ it is possible that bone‐derived growth factors released by acidification adsorb to the implant surface, thereby causing a cellular response during early osseointegration.

We have previously reported on acid bone lysate (ABL) containing transforming growth factor beta (TGF‐β)^15^ refining knowledge from the 1980s.^16^, ^17^, ^18^, ^19^ The concentration of TGF‐β1 with around 0.5 ng/mL in bone lysate is conserved among skeletal areas and gender.^16^ TGF‐β is released and activated by osteoclasts^20^, ^21^, ^22^ and supports migration of mesenchymal stem cells.^13^, ^23^ Recombinant TGF‐β1 adsorbs to Ti6Al4V decreasing osteogenic differentiation in vitro^24^ and adsorbs to TCP‐ceramic coated implants accelerating repair in a dog model.^25^ However, ABLs are a complex mixture of proteins that cannot be compared with recombinant TGF‐β.^15^ Thus, whether or not TGF‐β in ABL adsorbs to titanium implants causing a cellular response in vitro remains elusive.

Bioassays can determine a potential cellular response to implants exposed to ABL. We previously used oral fibroblasts to detect the TGF‐β activity in supernatants of freshly prepared bone chips,^12^ enamel matrix derivatives,^26^ and demineralized bone.^27^ Moreover, the adsorption of TGF‐β from these preparations to collagen matrices commonly utilized for guided bone regeneration was determined by bioassay.^28^, ^29^ The bioassay was based on whole genome microarray resulting in a panel of TGF‐β target genes including interleukin 11 (IL11), proteoglycan4 (PRG4), and NADPH oxidase 4 (NOX4).^30^, ^31^ Further support for the activation of TGF‐β signaling comes from phosphorylation and translocation of Smad3 into the nucleus.^32^

Since ABL prepared by the hydrochloric acid demineralization of bone chips activates TGF‐β signaling^15^ and that recombinant TGF‐β1 adsorbs to titanium alloys^24^ it was reasonable to suggest that bone‐derived TGF‐β binds to the titanium surface triggering a TGF‐β dependent cell response, thereby simulating the early stages of osseointegration.

## 2 MATERIALS AND METHODS

### 2.1 ABL and bone‐conditioned medium

Bone was obtained from adult pigs within 6 hours postmortem (Fleischerei Leopold Hödl, Vienna, Austria). Bone chips were harvested from the mandible with a bone scraper (Hu‐Friedy, Rotterdam, the Netherlands). Bone chips were washed with serum‐free Dulbecco's modified Eagle medium (DMEM) supplemented with antibiotics (Invitrogen Corporation, Carlsbad, California, USA). For ABL preparation, 5 g of wet bone chips were incubated while being stirred with 50 mL of 0.1 N HCl (10% weight/volume) at room temperature. ABL was harvested after 16 hours, centrifuged at 1200 RPM (358 g‐force) for 6 minutes and pH neutralized. After another centrifugation at 1200 RPM (358 g‐force) for 6 minutes, ABL was filtered sterile using a 0.2 μm syringe filter (VWR International, Pennsylvania, USA) and kept frozen at −20°C. The stocks were thawed immediately before each experiment. For bone‐conditioned medium (BCM) preparation, 5 g of bone chips were harvested from the mandible and incubated with 10 mL serum‐free culture medium DMEM (50% weight/volume) supplemented with antibiotics (Invitrogen Corporation). BCM was harvested after 24 hours of incubation at 37°C in a humidified atmosphere at 5% carbon dioxide. BCM was filtered sterile using a 0.2 μm syringe filter (VWR International, Pennsylvania, USA) and kept frozen at −20°C. The stocks were thawed immediately before each experiment.

### 2.2 Cell culture

Human gingiva was harvested from extracted wisdom teeth from patients who had given informed and written consent. An approval was obtained from the Ethics Committee of the Medical University of Vienna (EK NR 631/2007), Vienna, Austria. All experiments were performed in accordance with relevant guidelines and regulations. A total of three strains of fibroblasts were established by explant cultures and fewer than 10 passages were used for the experiments. ST2 mesenchymal stromal cells isolated from mouse bone marrow were obtained from RIKEN Cell Bank (Tsukuba, Japan), and were grown in Alpha‐MEM medium supplemented with 10% fetal calf serum (FCS; Invitrogen, Zug, Switzerland). For the differentiation experiments, the medium was supplemented with 50 μL/mL ascorbic acid (Invitrogen, Zug, Switzerland) and 10 mM β‐glycerophosphate (Invitrogen, Zug, Switzerland), as described previously.^12^ All cells used here were seeded at a concentration of 30 000 cells/cm^2^ onto the ABL‐coated titanium discs overnight. For all experiments two different titanium surfaces were tested, turned (Ti Gr. 4; Implacil De Bortoli, São Paulo, Brazil) and rough (TIO2‐blasted and acid‐etched; Implacil De Bortoli, São Paulo, Brazil). For coating the titanium discs, the discs were soaked for 1 hour either in ABL, BCM or serum‐free culture medium and then thoroughly washed three times with phosphate‐buffered saline for 1 minute. The inhibitor for the TGF‐β RI kinase, SB431542 (Calbiochem, Merck, Billerica, Massachusetts, USA) was used at 10 μM.

### 2.3 Cell viability

Titanium discs were exposed to ABL or serum free media for 1 hour and then washed with phosphate‐buffered saline for 1 minute. Thereafter, gingival fibroblasts were incubated onto the titanium discs overnight. MTT (3‐[4,5‐dimethythiazol‐2‐yl]‐2,5‐diphenyltetrazolium bromide; Sigma, St‐Louis, MO, USA) solution at a final concentration of 0.5 mg/mL was added to each well of a microtiter plate (CytoOne) for 2 hours at 37°C. The medium was removed and formazan crystals were solubilized with dimethyl sulfoxide. Optical density was measured at 570 nm. Data were expressed as percentage of optical density in the treatment groups normalized to unstimulated control values. In addition, cell viability was assessed by a Live‐Dead staining kit from Enzo Life Sciences AG (Lausen, Switzerland).

### 2.4 Reverse transcription‐PCR and immunoassay

Reverse transcription (RT) was performed with the SensiFAST cDNA Synthesis Kit (Bioline Reagents Ltd, London, UK). RT‐PCR was done with SensiFAST SYBR Kit using the manufacturer's instructions (Bioline). Amplification was performed with the StepOnePlus Real‐Time PCR System (Applied Biosystems, Life Technologies, Carlsbad, California, USA). Primer sequences are given in Table 1. Relative gene expression was calculated with the delta delta CT method. Reactions were run in duplicates. The supernatant was analyzed for IL11 using an immunoassay according to the manufacturer's instructions (R&D Systems, Minneapolis, Minnesota, USA).

**Table 1:** Primer sequences

	Sequence forward	Sequence reverse
hGAPDH	aag cca cat cgc tca gac ac	gcc caa tac gac caa atc c
hNOX4	tct tgg ctt acc tcc gag ga	ctc ctg gtt ctc ctg ctt gg
hIL11	gga cag gga agg gtt aaa gg	gct cag cac gac cag gac
hBMP2	cag acc acc ggt tgg aga	cca ctc gtt tct ggt agt tct tc
hBMP6	aca tgg tca tga gct ttg tga	act ctt tgt ggt gtc gct ga
hMMP10	cat acc ctg ggt ttt cct cca a	gtc cgc tgc aaa gaa gta tgt ttt c
hMMP13	tga gag tca tgc caa caa att c	cag cca cgc ata gtc atg tag a
hPAI1	aag gca cct ctg aga act tca	ccc agg act agg cag gtg
hCTGF	ctc ctg cag gct aga gaa gc	gat gca ctt ttt gcc ctt ctt
mGAPDH	aac ttt ggc att gtg gaa gg	gga tgc agg gat gat gtt ct
mALP	aac cca gac aca agc att cc	gag aca ttt tcc cgt tca cc
mOP	act cca atc gtc cct aca gtc g	tga ggt cct cat ctg tgg cat

### 2.5 Immunofluorescence

Immunofluorescent analysis was performed on human gingival fibroblasts plated onto titanium discs treated with ABL for 1 hour. Cells were fixed in paraformaldehyde and blocked in 1% BSA and 0.3% Triton in PBS at room temperature for 1 hour. Cells were subsequently incubated with Smad2/3 antibody (1:800, D7G7 XP Rabbit mAb #8685, Cell Signaling, Beverly, Massachusetts, USA) overnight at 4°C. Alexa Fluor 488 secondary antibody (1:500; Anti‐Rabbit, Cell signaling Technology, USA) was applied for 1 hour. Cells were washed and mounted onto titanium discs. Fluorescent images were captured at 40× using a fluorescent microscope (Oxion, Euromex, the Netherlands).

### 2.6 Statistical analysis

All experiments were repeated three to five times. Bars show the mean and standard deviation of the data from all independent experiments. Statistical analysis was based on Mann‐Whitney *U* test and Kruskal‐Wallis test with Dunn's multiple comparisons correction. Analyses were performed using Prism v7 (GraphPad Software, La Jolla, California, USA). Significance was set at *P* < 0.05.

## 3 RESULTS

### 3.1 Cell viability is maintained upon ABL coating of titanium

**Figure 1:**
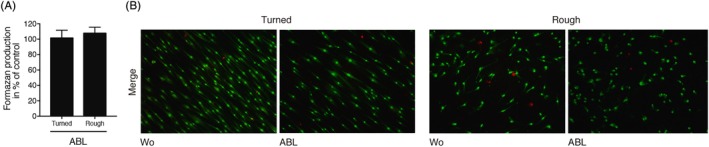
Cell viability is maintained upon acid bone lysate (ABL) coating of titanium. Turned and rough titanium discs were treated with ABL for 1 hour followed by three vigorous washes with buffered saline. Oral fibroblasts were seeded onto the discs for 24 hours before a A, MTT assay and B, a live‐dead staining was performed. Merged fluorescent images with living cells appearing in green and dead cells in red. Oral fibroblasts seeded on turned titanium discs displayed a more spindle‐shaped morphology in comparison to those seeded on rough discs. *N* = 4. Data represent the mean ± standard deviation relative to the control

To evaluate the impact of ABL coated discs on cell viability, the formation of formazan was determined. Exposure of titanium discs to ABL followed by extensive washing with buffered saline did not change the viability of human gingival fibroblasts on both titanium surfaces, turned or rough (Figure 1A). Cell viability was further confirmed by Live‐Dead staining demonstrating most notably high living cells (Figure 1B). These findings indicate that cells seeded onto titanium discs coated with ABL are viable under the present in vitro model.

### 3.2 Cells increase IL11 upon ABL coating of titanium

**Figure 2:**
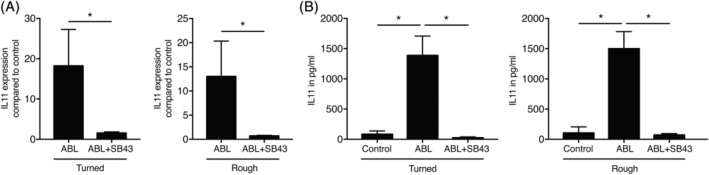
Cells increase IL11 upon acid bone lysate (ABL) coating of titanium. Turned and rough titanium discs were treated with ABL for 1 hour followed by three vigorous washes with buffered saline. Gingival fibroblasts were seeded onto the ABL coated titanium discs overnight with and without SB431542, the inhibitor for the TGF‐β RI kinase. A, Reverse transcription PCR analysis and B, immunoassays were performed for IL11. *N* = 3‐5. Data represent the mean ± standard deviation relative to the control of independent experiments. Mann‐Whitney *U* test (A) and Kruskal‐Wallis test with Dunn's multiple comparisons correction (B) were performed. Significance is indicated by *P < 0.05

We next investigated the cellular response to titanium discs exposed to ABL followed by vigorous washing with buffered saline. Gingival fibroblasts were seeded onto titanium discs with turned and rough surfaces and the expression of the TGF‐β target genes IL11 (Figure 2) and NOX4 (Supporting Information Figure S1) was determined as previously reported.^15^ Titanium surfaces upon ABL treatment, irrespective of their modification, caused a robust gene expression of IL11 (Figure 2A) and NOX4 (Supporting Information Figure S1) in gingival fibroblasts. The TGF‐β receptor I kinase inhibitor SB431542 blocked the respective increase in gene expression. Similarly, recombinant TGF‐β adsorbed to titanium discs as indicated by the increased IL11 and NOX4 expression (Supporting Information Figure S2). Figure 2B shows that also at protein level, ABL treatment of turned or rough titanium discs caused a robust SB431542‐dependent IL11 release into the cell‐culture supernatant. These results suggest that ABL holds a TGF‐β activity that adsorbs to titanium discs.

### 3.3 Cells activate TGF‐β‐Smad2/3 signaling upon ABL coating of titanium

**Figure 3:**
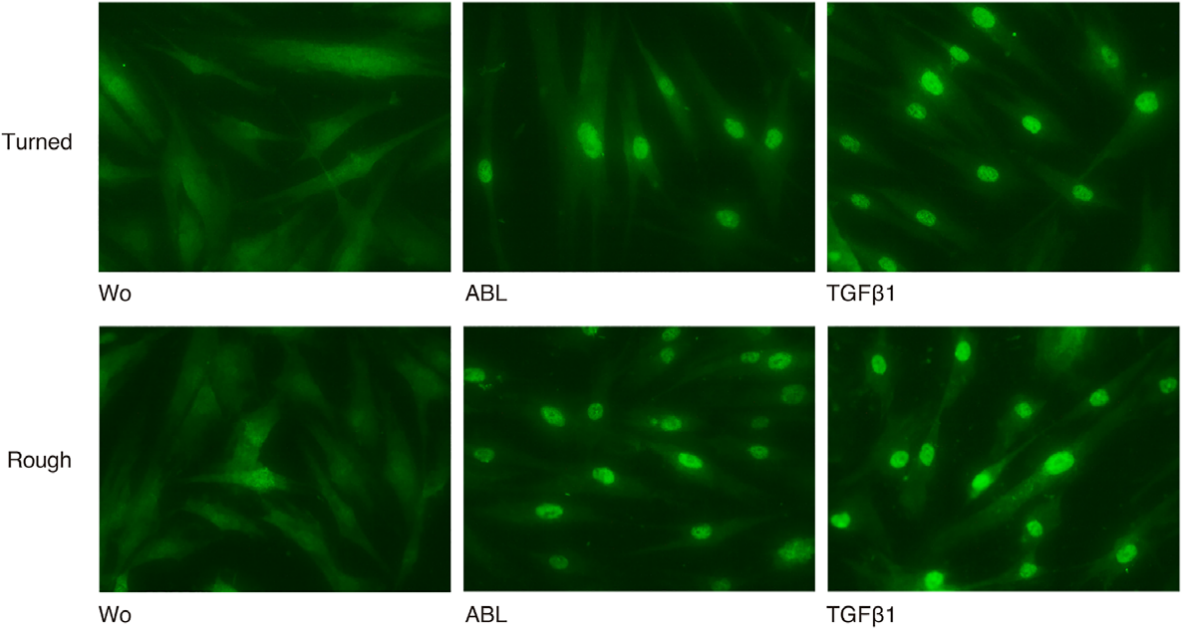
Acid bone lysate (ABL) activates TGF‐β‐Smad2/3 signaling in primary oral fibroblasts on titanium surfaces. Immunofluorescent analysis was performed on human gingival fibroblasts plated onto titanium discs treated with ABL for 1 hour followed by three vigorous washes with buffered saline. After 24 hours, cells were incubated with Smad2/3 antibody and detected with Alexa Fluor 488 secondary antibody. Representative immunofluorescence confirmed the translocation of Smad2/3 into the nucleus on titanium discs coated with either ABL or recombinant TGF‐β

To further verify the activation of TGF‐β signaling, immunofluorescent analysis of Smad2/3 nuclear translocation was performed. Fluorescent images revealed an indistinct signal when cells were seeded onto untreated titanium discs. However, a clear nuclear staining of Smad2/3 was visible with titanium discs coated with either ABL or recombinant TGF‐β (Figure 3). These findings further support the hypothesis that ABL‐derived TGF‐β activity adsorbs to titanium discs.

### 3.4 Cells increase IL11 and NOX4 upon BCM coating of titanium

**Figure 4:**
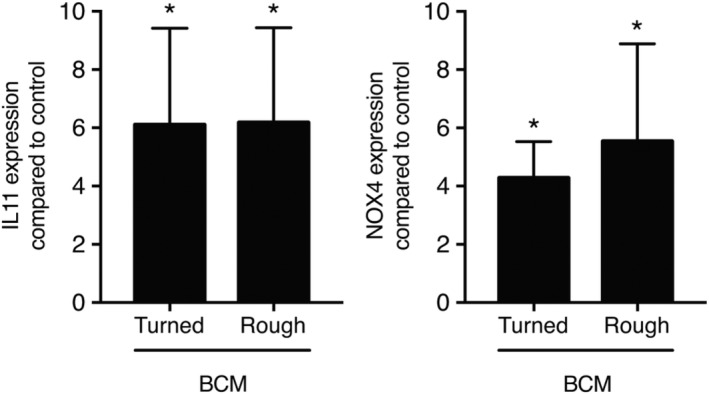
Cells increase IL11 and NOX4 upon BCM coating of titanium. Turned and rough titanium discs were treated with BCM for 1 hour followed by three vigorous washes with buffered saline. Gingival fibroblasts were seeded onto the BCM‐coated titanium discs for 24 hours. Reverse transcription PCR analyses were performed for IL11 and NOX4. *N* = 4. Data represent the mean ± standard deviation relative to the control of independent experiments. Mann‐Whitney *U* test was performed. Significant changes compared to unstimulated control discs are indicated by *P < 0.05

Inspired by previous research by our group where we showed that BCM holds a TGF‐β activity,^12^ gingival fibroblasts were seeded onto titanium discs that were soaked in BCM followed by vigorous washing with buffered saline. Similar to our findings with ABL, titanium discs incubated with BCM caused a robust expression of IL11 and NOX4 with no differences between the surfaces (Figure 4). These data suggest that TGF‐β activity, also when derived from native bone chips, adsorbs to titanium discs.

### 3.5 Cells increase other genes possibly relevant for osseointegration upon ABL coating of titanium

**Figure 5:**
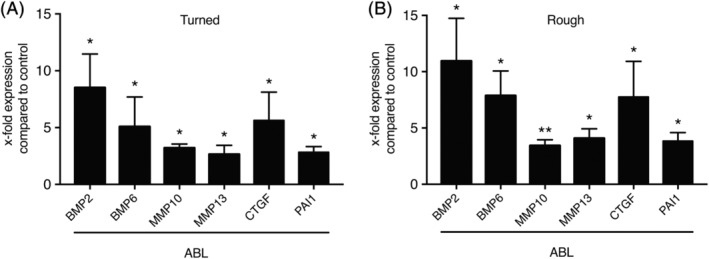
Target genes possibly relevant for osseointegration upon ABL coating of titanium. Turned (A) and rough (B) titanium discs were treated with ABL for 1 hour followed by three vigorous washes with buffered saline. Gingival fibroblasts were seeded onto the ABL coated titanium discs for 16 hours. Reverse transcription PCR analysis was performed for the indicated target genes. *N* = 4‐5. Data represent the mean ± standard deviation relative to the control of independent experiments. Mann‐Whitney *U* test was performed. Significant changes compared to unstimulated control discs are indicated by * *P* < 0.05 and **P < 0.01

We subsequently examined other potential TGF‐β target genes that possibly play a role during osseointegration of dental implants. Titanium surfaces upon ABL coating, regardless of their surface modification, significantly increased the expression of BMP2, BMP6, MMP10, MMP13, CTGF, and PAI‐1 (Figure 5). These findings further indicate that titanium discs can adsorb TGF‐β activity upon soaking in ABL, which then increases the expression of genes that are of potential relevance for the osseointegration of dental implants, including growth factors and proteases.

### 3.6 Cells decrease alkaline phosphatase activity upon ABL coating of titanium

**Figure 6:**
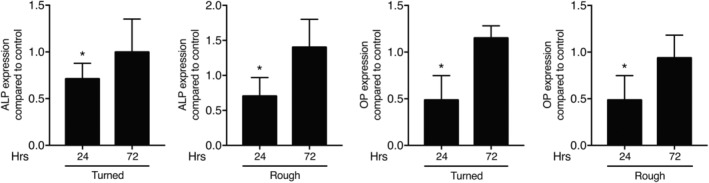
Cells decrease alkaline phosphatase activity upon ABL coating of titanium at an early stage. Turned and rough titanium discs were treated with ABL for 1 hour followed by three vigorous washes with buffered saline. ST2 cells were seeded onto the ABL‐coated titanium discs for 24 and 72 hours. Reverse transcription PCR analysis were performed for alkaline phosphate (ALP) and osteopontin (OP). *N* = 3‐4. Data represent the mean ± standard deviation relative to the control of four independent experiments. Mann‐Whitney *U* test was performed. Significant changes compared to unstimulated control discs are indicated by *P < 0.05

We next investigated whether, as reported for ABL and BCM, ST2 mesenchymal cells respond to ABL coating of titanium with a decrease in alkaline phosphatase^12^, ^15^ and osteopontin. In accordance with these studies, titanium discs incubated with ABL caused a significant decrease of alkaline phosphatase and osteopontin in ST2 cells at the level of gene expression within 24 hours of incubation (Figure 6). These findings suggest that ABL caused a transient inhibition of osteogenic differentiation.

## 4 DISCUSSION

Osseointegration is based on the fundamental principle that new bone grows on the surface of implants without a fibrous interface.^33^ Even though the clinical success of implant dentistry has developed constantly over more than three decades, the detailed molecular and cellular events leading to osseointegration are only beginning to be uncovered.^7^ Early suggestions based on histological images have coined the term “distance and contact osteogenesis” meaning that new bone can form on the surface of pristine bone but also on the surface of dental implants.^34^ Contact osteogenesis requires osteogenic cells to migrate toward the implant surface where they adhere and initiate the production of osteoid that is later reinforced by minerals.^34^ There is also evidence that serum components adsorbing to the implant surface enhance a biological activity for the arriving osteogenic cells.^11^ Apart from serum components, bone can release molecules, either passively upon preparation of an implant bed^11^, ^31^ or via osteoclasts during bone resorption.^13^ However, there are currently no studies showing if native bone‐derived growth factors can adsorb to an implant surface and cause a biological response.

The results of the current study showed that TGF‐β activity of acid‐bone lysates^15^ adsorbs to titanium implant surfaces. This TGF‐β activity was determined by a bioassay showing the expression of the respective target genes, including IL11 and NOX4, but also BMP2 and BMP6, both being growth factors relevant for bone regeneration^35^, ^36^ and MMP10, MMP13, and PAI‐1, together regulating the remodeling of extracellular matrix.^37^, ^38^ Also, CTGF being among the target genes, is critically involved in wound healing and in fibrotic pathologies.^39^, ^40^ Moreover, our results revealed that the binding of ABL activity was independent of the surface modification suggesting that the advantages of rough surface implants over turned implants^41^ cannot be explained by differential TGF‐β activity binding. These data suggest that bone‐derived TGF‐β activity adsorbs to titanium surfaces presumably during the first days of osseointegration where bone formation on the surface of the implants becomes visible.^1^

If we relate these findings to those of others, recombinant TGF‐β1 adsorbs to Ti6Al4V^24^ and ceramic‐coated implants.^25^ It is also well accepted that bone contains TGF‐β,^16^, ^17^, ^18^, ^19^ and we have recently extended this knowledge by confirming the TGF‐β activity for oral cells.^15^ This knowledge, however, does not necessarily mean that TGF‐β activity of ABL exclusively binds to titanium and resists extensive washing. Even though bone contains BMPs^42^, ^43^ and recombinant BMPs can adsorb to titanium,^44^, ^45^ we have not detected BMPs in ABL by proteomic analysis.^15^ Thus, bone‐derived BMPs might adsorb to titanium but the concentration is below the threshold that causes a cellular response such as activation of the BMP‐target gene ID2 (data not shown) and the osteogenic differentiation markers alkaline phosphatase and osteopontin. Thus, bioassays revealed bone‐derived TGF‐β but not BMP activity adsorbing to titanium.

The present findings provide a basis for future research. Considering that in vivo, TGF‐β attracts mesenchymal cells which later become osteoblasts during bone remodeling^46^ and that TGF‐β supports the formation of an extracellular collagen‐rich matrix but prevents early osteogenic differentiation,^47^ the binding of TGF‐β activity might be crucial for the early stages of osseointegration and likely also for graft consolidation. Our study is a first attempt toward this research direction and several questions remain to be answered. For example, to what extent can TGF‐β released during peri‐implant bone‐resorption in vivo contribute to contact osteogenesis and what is the major cellular response? It is unlikely that the genes we have selected substantially contribute to osseointegration as they were selected mainly based on the sensitivity of TGF‐β signaling rather than to control cellular behavior during osseointegration. The present research raises even further questions.

How can the observations be interpreted with respect to peri‐implantitis, where inflammatory osteolysis causes the release of TGF‐β1 from bone? Under the pathological conditions, extensive release of TGF‐β1 from bone but also other sources might contribute to exceeding collagen production and thus fibrosis that could culminate into implant loss–a hypothesis that remains to be tested. Moreover, the inflammatory environment in peri‐implantitis is presumably catabolic and suppresses bone formation.^48^ Thus, the in vitro setting presented here does not reflect the pathological situation of peri‐implantitis. Also, the role of other adsorbed proteins than TGF‐β have not been covered in the present study. Our research included serum components, as we have seeded cells on the ABL‐exposed discs in the presence of serum‐containing medium. This in vitro setting, however, does not rule out the distinct roles of plasma components on osseointegration in vivo, which are represented by platelet‐rich fibrin. In our recent research, we can confirm the binding of TGF‐β activity on titanium discs. If, however, the plasma and the bone‐derived TGF‐β activity competes for the binding to titanium, and if plasma components even inhibit the absorption of bone‐derived TGF‐β remains to be determined.

The target cells and their origin are another issue leading to speculations. Even though the origin of the mesenchymal cells in vivo that become osteoblasts in contact osteogenesis needs to be determined, it is likely that they are derived from H‐type capillaries that form as a consequence of angiogenesis.^49^ According to our observations that ABL suppresses osteogenic differentiation of ST2 mesenchymal stromal cells, it is possible that also in vivo, early osteogenic differentiation is delayed until the catabolic translates into an anabolic environment. Our data showing that the TGF‐β activity adsorbed to titanium fades after 24 hours support this assumption (Supporting Information Figure 3). Moreover, mesenchymal cells might be one of several potential TGF‐β target cells that are involved in osseointegration. There is room for research identifying the response of other lineages such as the monocytes and their differentiation and polarization toward the inflammatory or resolving phenotypes, or into bone‐resorbing osteoclasts.

With regards to the research methods, there are also limitations that need to be acknowledged. Firstly, ABL only to some extent simulates the situation after implant insertion as TGF‐β activity is also passively released from injured bone^12^, ^50^ and osteoclasts not only use acids, but also proteases such as cathepsin K to digest bone matrix.^51^ Secondly, it remains a hypothesis whether bone‐derived TGF‐β adsorbing to titanium plays a role in early osseointegration and if so, to what extent? Studies are needed where implants are placed in bone lacking TGF‐β1, or at least where target cells including those of the mesenchymal lineage lack the corresponding TGF‐β receptor.^52^ Moreover, there are other sources of TGF‐β than the bone and their contribution to osseointegration are also a basis for future research. Thirdly, our finding that surface modification does not affect the cellular response to ABL in vitro does not necessarily explain the in vivo situation. Fourthly, the adsorption kinetic of bone‐derived TGF‐β activity under different conditions such as temperature, time and long‐term storage should be determined further. One option would be that implant surfaces can be modified with the aim to control the TGF‐β binding, in particular because TGF‐β might suppress bone formation and contribute to scar formation.^53^ Fifthly, it is unknown if implants made from zirconia show a similar affinity for bone‐derived TGF‐β activity. Sixth, the question if TGF‐β on the titanium surface is capable to attract mesenchymal cells is a research opportunity. Finally, it would be interesting to determine the impact of implants coated with ABL on the early stages of osseointegration in a preclinical study.

Our findings that bone‐derived TGF‐β activity rapidly adsorbs to titanium may provide a new piece of the puzzle in our understanding of the early stages of osseointegration. These in vitro data may be an inspiration for future research on how bone‐derived molecules contribute to the process of osseointegration with the overall clinical goal to consider these mechanisms in surgical techniques and in the development of implant‐related biomaterials.

## CONFLICT OF INTEREST

The authors declare no conflict of interest.

## Supporting information


**Supplemental Figure 1** *Cells increase NOX4 upon ABL coating of titanium*
Turned and rough titanium discs were treated with ABL for 1 hour followed by three vigorous washes with buffered saline. Gingival fibroblasts were seeded onto the ABL‐coated titanium discs for 16 hours with and without SB431542, the inhibitor for the TGF‐β RI kinase. Reverse transcription PCR analysis was performed for NOX4. N = 3‐5. Data represent the mean ± SD relative to the control of independent experiments. Mann‐Whitney U test was performed. Significance is indicated by * *P* < 0.05

**Supplemental Figure 2** Cells increase IL11 and NOX4 upon TGF‐β1 coating of titanium
Turned and rough titanium discs were treated with TGF‐β1 for 1 hour followed by three vigorous washes with buffered saline. Gingival fibroblasts were seeded onto the coated titanium discs for 16 hours. Reverse transcription PCR analysis was performed for IL11 and NOX4. N = 3‐5. Data represent the mean ± SD relative to the control of independent experiments

**Supplemental Figure 3** *Cells increase IL11 upon ABL coating of titanium*
Turned and rough titanium discs were treated with ABL for 1 hour followed by three vigorous washes with buffered saline. After 24, 48 and 72 hours, gingival fibroblasts were seeded onto the ABL‐coated titanium discs for 24 hours and ELISA for IL11 was performed
