# Supplementary figures and images for: TGF‐β activity in acid bone lysate adsorbs to titanium surface
Source: Clin Implant Dent Relat Res. 2019 Feb 28;21(2):336–43. doi: 10.1111/cid.12734 (PMC6593995; doi:10.1111/cid.12734)

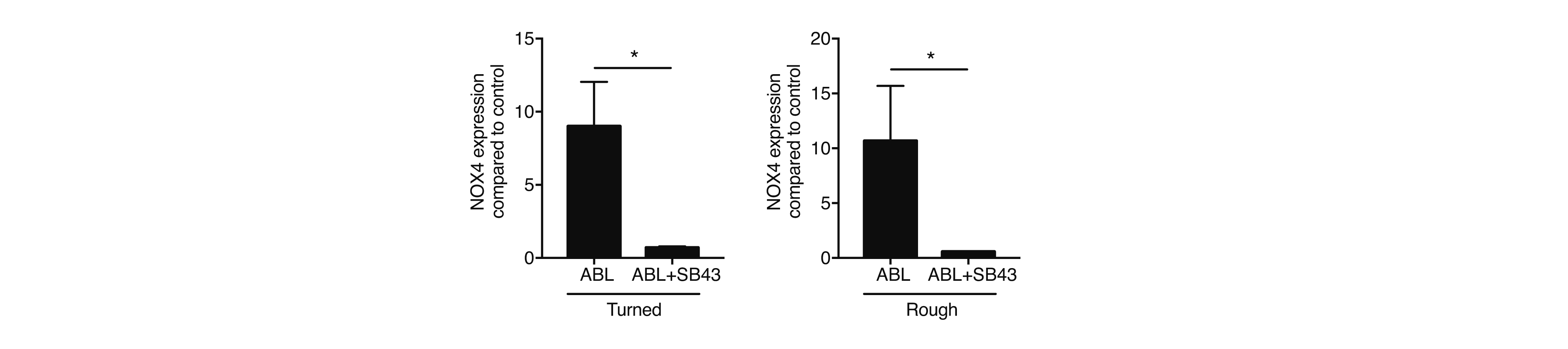

Supplement: Supplementary file 1 — Supplemental Figure 1 Cells increase NOX4 upon ABL coating of titanium Turned and rough titanium discs were treated with ABL for 1 hour followed by three vigorous washes with buffered saline. Gingival fibroblasts were seeded onto the ABL‐coated titanium discs for 16 hours with and without SB431542, the inhibitor for the TGF‐β RI kinase. Reverse transcription PCR analysis was performed for NOX4. N = 3‐5. Data represent the mean ± SD relative to the control of independent experiments. Mann‐Whitney U test was performed. Significance is indicated by * P < 0.05 [file CID-21-336-s001.tif]

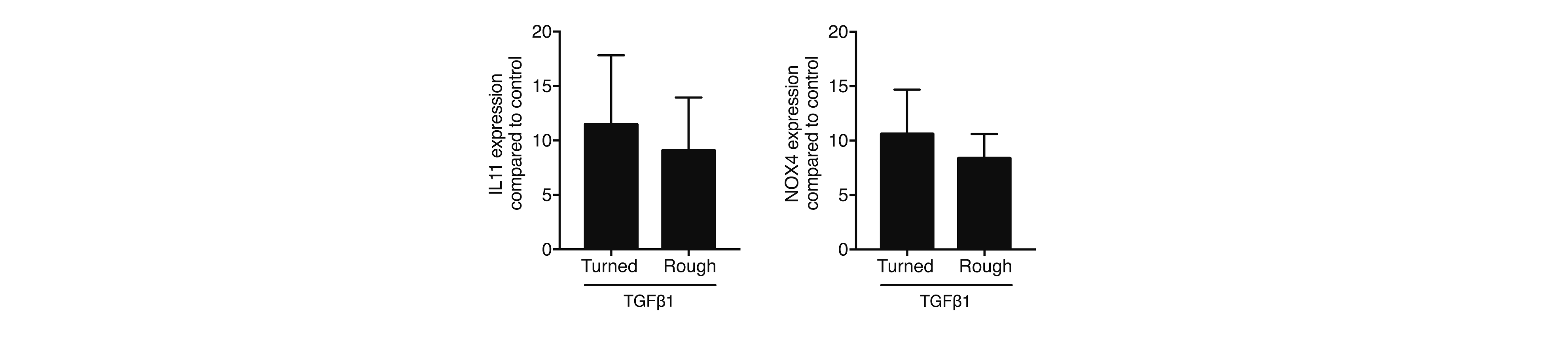

Supplement: Supplementary file 2 — Supplemental Figure 2 Cells increase IL11 and NOX4 upon TGF‐β1 coating of titanium Turned and rough titanium discs were treated with TGF‐β1 for 1 hour followed by three vigorous washes with buffered saline. Gingival fibroblasts were seeded onto the coated titanium discs for 16 hours. Reverse transcription PCR analysis was performed for IL11 and NOX4. N = 3‐5. Data represent the mean ± SD relative to the control of independent experiments [file CID-21-336-s002.tif]

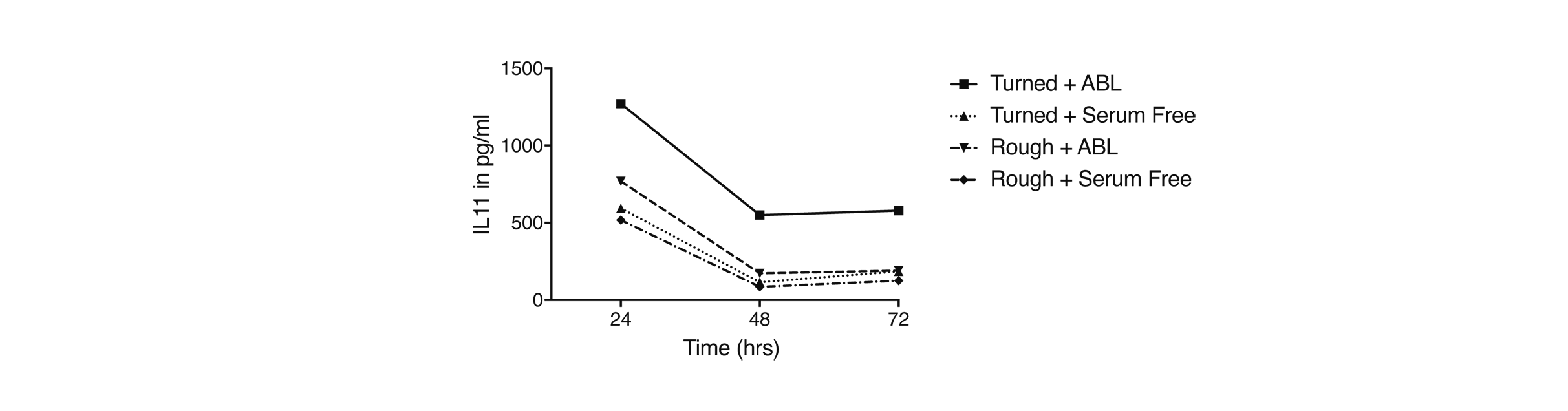

Supplement: Supplementary file 3 — Supplemental Figure 3 Cells increase IL11 upon ABL coating of titanium Turned and rough titanium discs were treated with ABL for 1 hour followed by three vigorous washes with buffered saline. After 24, 48 and 72 hours, gingival fibroblasts were seeded onto the ABL‐coated titanium discs for 24 hours and ELISA for IL11 was performed [file CID-21-336-s003.tif]
